# Impact of Preventive Responses to Epidemics in Rural Regions

**DOI:** 10.1371/journal.pone.0059028

**Published:** 2013-03-11

**Authors:** Phillip Schumm, Walter Schumm, Caterina Scoglio

**Affiliations:** 1 K-State EpiCenter, Department of Electrical and Computer Engineering, Kansas State University, Manhattan, Kansas, United States of America; 2 Department of Family Studies and Human Sciences, Kansas State University, Manhattan, Kansas, United States of America; Glaxo Smith Kline, Denmark

## Abstract

Various epidemics have arisen in rural locations through human-animal interaction, such as the H1N1 outbreak of 2009. Through collaboration with local government officials, we have surveyed a rural county and its communities and collected a dataset characterizing the rural population. From the respondents’ answers, we build a social (face-to-face) contact network. With this network, we explore the potential spread of epidemics through a Susceptible-Latent-Infected-Recovered (SLIR) disease model. We simulate an exact model of a stochastic SLIR Poisson process with disease parameters representing a typical influenza-like illness. We test vaccine distribution strategies under limited resources. We examine global and location-based distribution strategies, as a way to reach critical individuals in the rural setting. We demonstrate that locations can be identified through contact metrics for use in vaccination strategies to control contagious diseases.

## Introduction

In general, the spread of infectious diseases can be contained by human response using different approaches. Susceptible people can acquire immunization through vaccination, or can protect themselves from the diseases using preventive behaviors, such as avoiding close physical contacts with infected individuals or using hygienic habits. Correspondingly, human responses can be modeled using three classes of models distinguished by changes taking place in compartments, parameters, or contact levels to take into account the behavioral changes [1].

A vast literature exists on efficient vaccination strategies, given the need for efficient strategies to distribute vaccines that can often be insufficient for the entire population. Some of these strategies assume that human contact networks are well represented by scale free networks. One popular strategy aims at immunizing those individuals having the highest number of contacts, as the most critical actors for spreading the infection [2]. However, local strategies are more efficient and implementable and often require a lower fraction of the population to be vaccinated than random global immunization to contain epidemics. The strategy of acquaintance immunization proposes the immunization of random acquaintances of random individuals [3]. Another local strategy proposes to vaccinate highly connected acquaintances of randomly selected people; based on the properties of scale free networks, with this approach the probability of targeting the highly connected individuals in the contact network increases with respect to the simple random selection [4]. In the case of a limited amount of available vaccines, the authors of [5] use stochastic simulations of epidemic and numerical optimization methods to find near-optimal vaccine distributions to minimize the epidemic size. Again in the case of a limited amount of available vaccines, the best strategy suggests to vaccinate schoolchildren, the population group with highest contact in different communities, and the high-risk groups, the population groups that need protection [6]. Since a strong community structure can be detected in social contact networks, the approach in [7] aims at immunizing individuals bridging communities rather than simply targeting highly connected individuals. An extensive set of simulations performed in [8] suggests two strategies based on age classes: In the first strategy vaccinating older children, adolescents, and young adults minimizes the number of infections, while in the second strategy vaccinating either younger children and older adults or young adults minimizes the number of deaths. Using game theory, the authors of [9] show that when vaccination is an individual’s choice, a periodic behavior can be seen in simulations. A severe epidemic in one year incentivizes high vaccination rates in the following year, causing a milder epidemic for which individuals have less motivation for vaccination in the subsequent year. In [10], authors develop a vaccination strategy based on optimizing the susceptible size by a partitioning of the contact network through vaccination. Based on the authors’ simulations, this strategy is more efficient than those based on vaccinating the highest betweenness or contact individuals. Using a decision-making framework for vaccine distribution policies based on a geographical and demographical data in USA, the authors of [11] find that distributing vaccines first to counties where the latest epidemic waves are expected is the most efficient policy.

In any case, assessing the effectiveness of mitigation strategies and behavioral responses both from a public health point of view and from individuals’ perspectives is a complex and not fully-explored problem. In particular, a thorough evaluation and comparison of feasible mitigation strategies in the specific setting of rural regions is missing. In other words, not only the amount of success a given strategy can provide is not determined, but also its related cost in economical and social terms is unknown.

In this paper, we carry out extensive simulations on a weighted contact network determined by collected data in the City of Chanute and Neosho County in the State of Kansas. In particular we study the impact of limited resource vaccination campaigns, using an exact model of a stochastic SLIR Poisson process. Simulations are run across several scenarios and with stochastic sets of the SLIR model parameters. The evaluation of the vaccination campaigns is performed computing the average number of cases prevented per a single vaccine and the sizes and durations of the outbreaks. Our contributions are twofold: we construct and analyze a data-based rural contact network and we provide a thorough analysis and comparison of mitigation strategies in a rural region. We hope that our results can provide practical guidelines for health officials to contain and suppress epidemics in rural regions.

## Methods

In the following we describe the data collection and analysis, and the models for the network, for the epidemic spreading, and also for vaccination strategies and distributions.

### 1. Data Collection and Analysis

As of the 2010 U.S. Census, Neosho County was a rural county with 16,512 residents in 571.5 square miles in southeastern Kansas. Most of the population was White (94.1%); a majority were female (50.6%) and many (17.4%) were 65 years of age or older. The median household income was $36,702 with 17.0% living below the poverty level. Between July and October 2010, the towns of Chanute, Thayer, and Galesburg were selected to participate in a survey concerning factors that would predict the spread of epidemics in rural areas. From county public household rosters, households were randomly selected from Chanute (10%, N = 171), Thayer (50%, N = 158), and Galesburg (50%, N = 73) for a total initial *N* = 402. After considerations mentioned in the supplementary information, the final number of available and eligible households were 143, 65, and 162 in Thayer, Galesburg, and Chanute, respectively, with total *N* = 370.

The tailored design method was used, with minor modifications, to improve response rates [12]–[15] with a focus on personalization and multiple follow-up mailings. The initial survey also included a local news report announcing the impending start of the survey [16]. Overall, 242 surveys were for an overall response rate of 65.4%. The response rate for Chanute was 74.7% (121/162). The response rate for Thayer and Galesburg combined was 55.8% (116/208). The difference in response rates (74.7% vs. 55.8%) between Chanute and Thayer/Galesburg was significant statistically, two-sided Fisher’s Exact Test (*p*<.001), odds ratio = 2.34 (95% CI, 1.50–3.66, *p*<.001). The difference in response rate for the more urban location was probably related to the content of the survey, which focused on respondents’ visiting locations of stores, public sites, and restaurants in Chanute itself. Thus, the survey probably seemed more relevant to Chanute residents, even though we were interested in how often households from outlying towns went to the nearest urban center to visit or shop.

A majority (56%) of the respondents reported being from Chanute compared to 23% from Thayer and 10% from Galesburg (the remaining percentage did not specify exactly where they were from). Of the 357 participants, the largest number were ages 45 to 64 (47.1%), with 26.1% 65 years of age or older and 18.8% (26–44) and 8.1% (18 to 25) younger than 45. A majority of the participants were females (57.6%). Most of the respondents (75.4%) had lived in their local community for 15 years or more. The vast majority (97.5%) of the respondents lived in a single family home. Very few (6.2%) of the households included a homebound member. Most of the respondents had either the equivalent of a high school degree (22%) or a college (23%) or graduate (12%) degree. Nearly sixty percent had incomes between $25,000 and 100,000 a year with 11% earning more and 30% earning less. Some respondents had type I (1.2%) or type II diabetes (10.4%) or were pre-diabetic (3.2%). Most respondents considered themselves to be slightly (35.7%), somewhat (18.2%), or extremely (8.6%) overweight. Most (56.6%) reported that they ate out one or two times a week with 26% eating out more often and 17% not at all.

**Figure 1:**
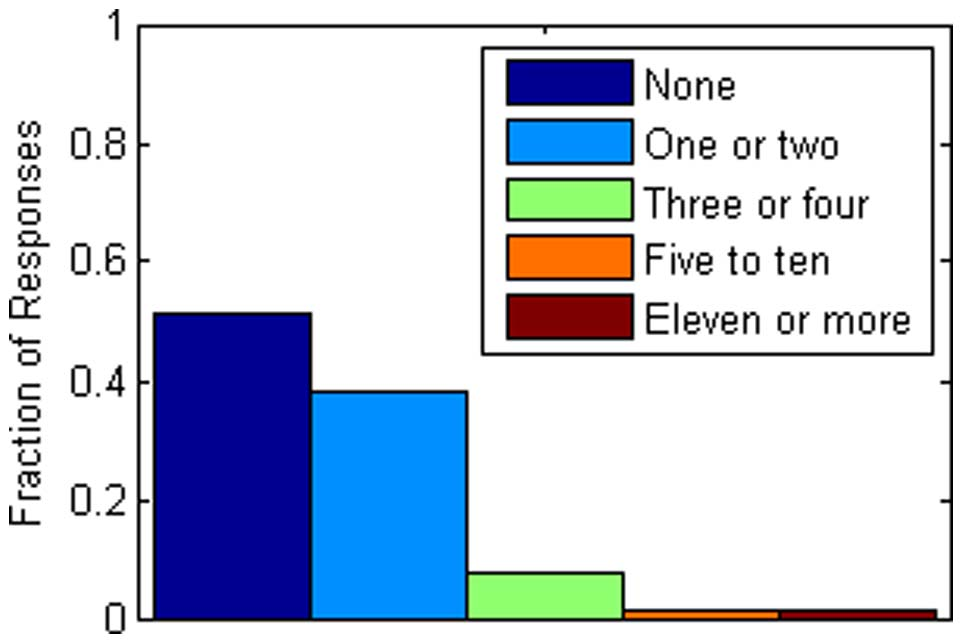
Distribution of discouraged household visits. The distribution of the number of households that a respondent expects to still visit in a week against advice during a serious epidemic is shown.

**Figure 2:**
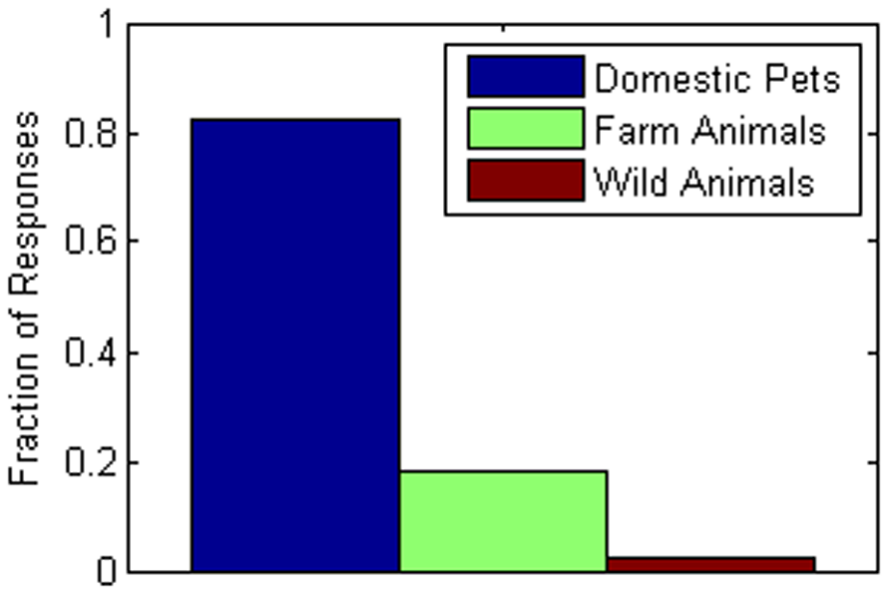
Distribution of human-animal interactions. The distribution of types of animals a respondent interacts with in a typical day is shown. Note that the total does not sum to one as respondents can interact with multiple types of animals.

In terms of compliance risk, nearly 49% of respondents said they would still visit at least one or two households outside of their home if there was a serious epidemic and radio/TV/internet had told them to remain at home and not visit with others. Figure 1 presents the distribution of the number of individuals that a respondent expects to still visit against advice. Only half (50.0%) of the respondents had been vaccinated against the flu within the past six months. Nearly 40 percent (38.9%) did not obtain such a vaccination because of concerns about the vaccine’s safety or effectiveness. Only about 7% believed they had come down with the flu within the past six months while about 18% thought they might have come down with a cold. About 18% of the respondents reported taking vitamin D supplements; only 6% reported taking zinc supplements. Approximately 80% of the respondents had extensive contact with domestic pets on a daily basis while about 19% of respondents had contact with farm animals or wild animals regularly, shown in figure 2. Contact risk (low, moderate, high) was significantly related statistically with compliance risk (none, low, high) (*p*<.001, ES = 0.50, medium effect size). As contact risk increased from low to high, high compliance risk increased from 4.4% to 21.8%; as contact risk decreased from high to low, the percentage of respondents with no compliance risk increased from 38.6% to 62.3%.

### 2. Models

Here, the procedure to construct the contact network from survey data is explained. Furthermore, the compartmental model used for simulations and the preemptive vaccination strategies are described.

From the survey responses, we constructed a rural contact network as an estimation of the social contact structure among the survey respondents. The network is based on two central questions: the number of contacts that a person has, and the locations that a person visits at different times in a typical day. The basis for the interactions between a pair of respondents is the locations that they both visited in common. We considered 4 types of location-based interactions: both visit the same location in the morning, both visit the same location in the afternoon, both visit the same location in the evening, and both visit the same location regardless of time. The fourth category introduces some overlapping in the interactions, but it is added to account for some of the uncertainty in potential pathways of the disease spread. We considered 66 locations in the network construction and therefore 264 = 66×4 possible interactions between each pair of survey respondents. We compute normalized weights from each respondent *i* to each other respondent *j* given by *l_ij_*, representing the number of location-based interactions between respondents *i* and *j*. For the few respondents who did not complete the section of the survey regarding location visits, we assign them uniform weights of interacting with every other respondent in the network. Letting nodes represent the set of *N* = 353 respondents and weighted links represent the contact between them; we have a symmetric contact network at this point. Next we uniformly scale the weights on the links directed outward from each respondent *i* such that the sum of these weights is equal to the number of contacts that respondent *i* has indicated having with his or her response (*w_ij_* = a_i_*l_ij_ for every *j* in 1, 2, … *N*). (This scaling makes irrelevant the absolute value of the uniform weight of the respondents who lack location data.) The bipartite network of locations and survey respondents is finally represented as a weighted, directed (asymmetrical), and unipartite contact network of 353 nodes, with each pair of nodes (*i* and *j*) connected by two links which are respectively characterized by the weights *w_ij_* and *w_ji_*.

**Figure 3:**
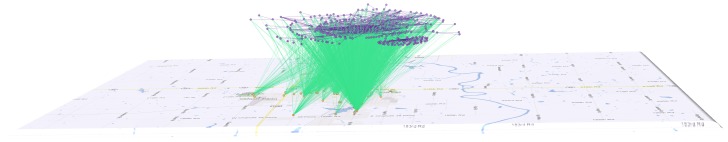
Survey-based rural contact network of Neosho County. A depiction of the rural contact network developed from a survey of Neosho County is shown, where the individuals are represented by purple nodes in a “cloud,” which is connected by the respondents local travel habits to the set of rural locations shown in orange on the map.

Six of the vaccination strategies will center on three node metrics: incoming node strength (the sum of the weights incoming to a node), outgoing node strength (the sum of the weights outgoing from a node), and node betweenness (a count of the shortest paths among all pairs that utilize the node) [17], [18]. The incoming node strength of a node is a topology metric that captures the direct impact of the network on the node. The outgoing node strength captures the direct impact that a node can have on the network. The betweenness of a node is a measure which captures the significance of a node in traversing the network. A node with a higher betweenness would be more likely to be traversed (in a shortest-path-type travel across the network between any pair of nodes) than a node with lower betweenness. Although an epidemic is not restricted to following the shortest paths across a network, the betweenness metric still plays an important role in identifying nodes which are likely to catch the disease if it reaches a majority of the nodes in the network. The rural contact network is depicted in figure 3, where the nodes representing individuals are shown in purple in a cloud and they are connected to the locations that they frequent, shown as orange nodes on the map [19].

On this weighted network, we model an epidemic outbreak using a Susceptible-Latent-Infected-Recovered compartmental model (SLIR) [17], [20]. In the SLIR model, we assume infections arrive at a susceptible (S) node *j* from an infected (I) node *i* with a rate that is a product of the directed contact *w_ij_* and the basic infection rate *β*. When an infection arrives to a susceptible node, the node takes on a latent infection (transfers from the susceptible compartment to the latent compartment). A node, once latent (L), is considered unable to spread the disease, but is developing to that stage with rate *λ*. The inverse of the rate *λ* is the expected time for a node to spend in the latent state. The next stage of the disease, the infected/infectious state, enables the node to spread infections to each of its neighbors at rates proportional to the weights on its outgoing links. Each infected node recovers from the infected state at a rate *µ*. Once a node is in the recovered state (R), it remains recovered and does not participate in the disease process any further.

We simulate this model exactly using an event-driven simulation of the SLIR process on the weighted rural contact network. We initialize the simulation by assigning a disease state to each node and then drawing exponential waiting times for the next event at each node. Taking the event with the minimum time across all nodes, we advance the event node to its next disease state and re-draw waiting times for all nodes. This step is repeated until all waiting times are infinite, which happens when the disease process is complete. At this point, all nodes will be either susceptible or recovered. In the event-driven simulation, the time periods between successive events will not be regular, but instead they are non-integer stochastic values.

**Figure 4:**
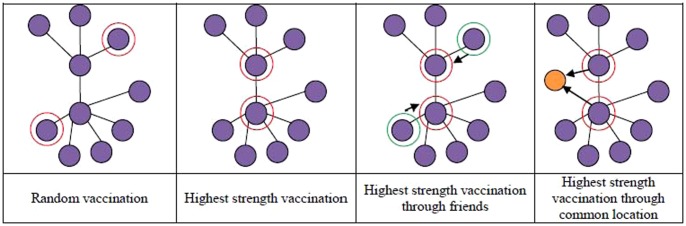
Example of vaccination strategies for individuals (purple nodes) in a contact network. Orange nodes represent locations, red circles represent the selected nodes for vaccination, and the green nodes represent random selected individuals, whose friends will be candidate for vaccination.

Vaccination is carried out by selecting a set of nodes and immunizing them with a certain vaccine efficacy rate. We consider seven different strategies for selecting the set of nodes for vaccination. The first and simplest strategy is a random selection of 10% of the population (35 nodes). The random method represents a blind distribution across the population. The next three strategies consider a targeted selection of nodes (individuals) based respectively on the three node metrics, incoming node strength, outgoing node strength, and node betweenness. These three strategies are idealistically implemented by selecting the 35 nodes with the highest values for the respective metric and administering the vaccine. For less ideal situations, we consider three additional strategies that attempt to represent feasible vaccine distribution strategies for rural populations. Considering again the three above mentioned network metrics, we determine the location which has the highest average value (on the set of nodes that visit the location) of each metric. These locations are a restaurant (outgoing node strength), a pharmacy (node betweenness), and a location used for public events (incoming node strength). After selecting the locations that represent on average the best places to find nodes with higher values of each metric, we consider a random selection within a location of 10% of the entire population for vaccination. This location-based targeting has been proposed in [20]. It allows an indirect (and thus more feasible) targeting of critical populations that ensures a more effective use of resources than widely distributing resources in a global manner. Note that there is an implicit assumption that the entire population is susceptible previous to the distribution of the vaccine. Although this is not a realistic assumption for a commonly occurring strain of influenza, it would likely be the case for any new disease threat. In figure 4, a simple exemplification of these strategies is described.

## Results

**Figure 5:**
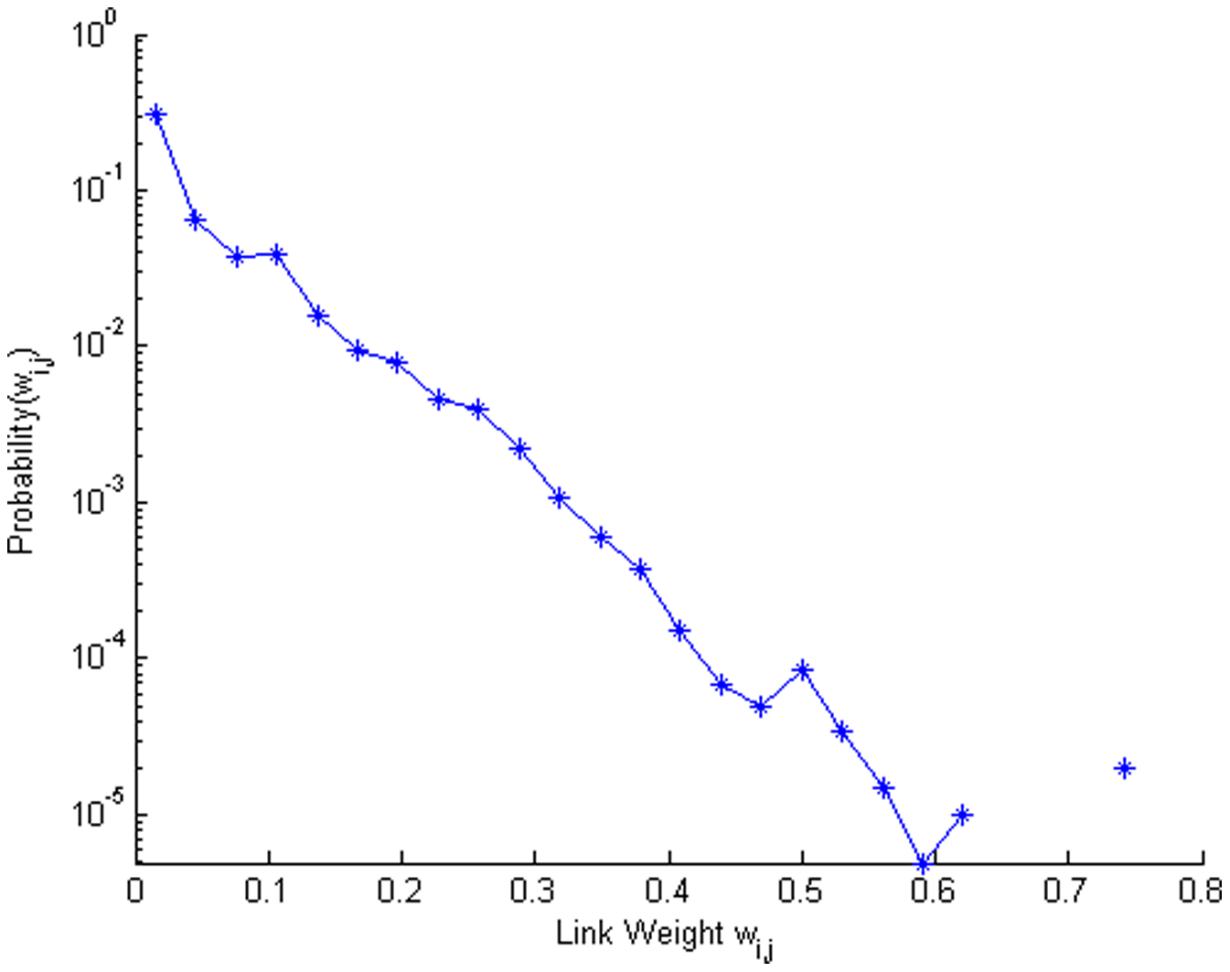
Distribution of the weights representing social contact for the rural community contact network. Note that the vertical axis has a log scale.

**Figure 6:**
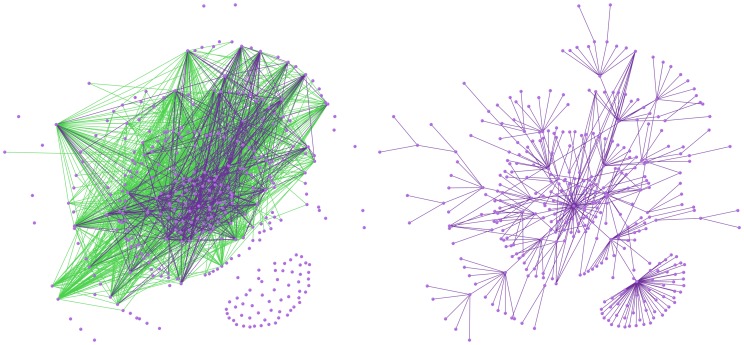
Two visualizations of the rural contact structure within the network. (Left) A visualization of the rural community contact network showing the nodes and the links having weights between 0.2 and 1.0, where the weights of the green links are between 0.2 and 0.3 and those of the purple links are between 0.3 and 1.0. (Right) A visualization of the rural community contact network showing the nodes and the links having weights between 0.4 and 1.0 as well as the “best-friends” links, where the best friend link of a node is defined as the link having the highest out-going weight.

We measured on the network the metrics of interest for the vaccination targeting strategies. Figure 5 shows the diversity found in the weights that measure the levels of contact between each neighboring pair of nodes. Roughly 30 percent of the links carry very small weights, and there are very few links representing the highest weighted contacts. In figure 6 we display two views of the network topology to visualize the estimated rural community contact structure. Since the network is rather dense, we remove the links with lower weights in two different patterns. On the left side of figure 6, we colored the nodes and the links having weights between 0.2 and 1.0, where the weights of the green links are between 0.2 and 0.3 and those of the purple links are between 0.3 and 1.0. In this depiction, a minority but significant set of individuals (roughly 50 nodes) can been noticed for their state of isolation. These nodes are not strongly connected to the core of the network, but are connected when the links with the lowest weights are considered. This loosely connected “fringe” of the rural community is rarely reached by epidemics until a very strong epidemic comes. On the right side of figure 6, we colored the nodes and the links having weights between 0.4 and 1.0 as well as what we call the “best-friends” links. For each node, we select the link having the highest out-going weight and define this link as the “best-friend” link of the node. This depiction of the network captures the most likely paths (it is composed of the highest weighted links) that an epidemic might take from anywhere in the network towards the center of the network. Although this pattern of visualization may give the false impression that the network is tree-like or scale-free, an epidemic would leave a tree-like pattern as it traces its way through the rural community. Note that figure 5 proves that both of these network visualizations in figure 6 are missing majorities of the links in the complete network.

**Figure 7:**
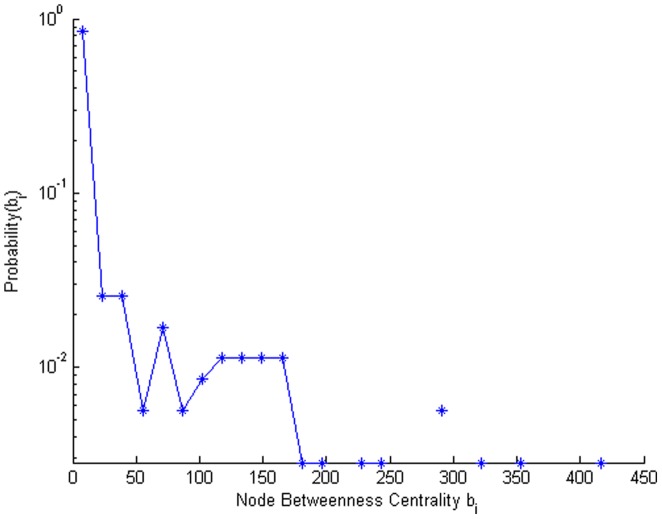
Distribution of the node betweenness values of the individuals in the rural community contact network. Note that the vertical axis has a log scale.

Figure 7 shows the distribution of the node betweenness metric for the network. More than 80 percent of the nodes have very small values of node betweenness, leaving a select group of nodes that are critical connections in the system of shortest paths through the community.

**Figure 8:**
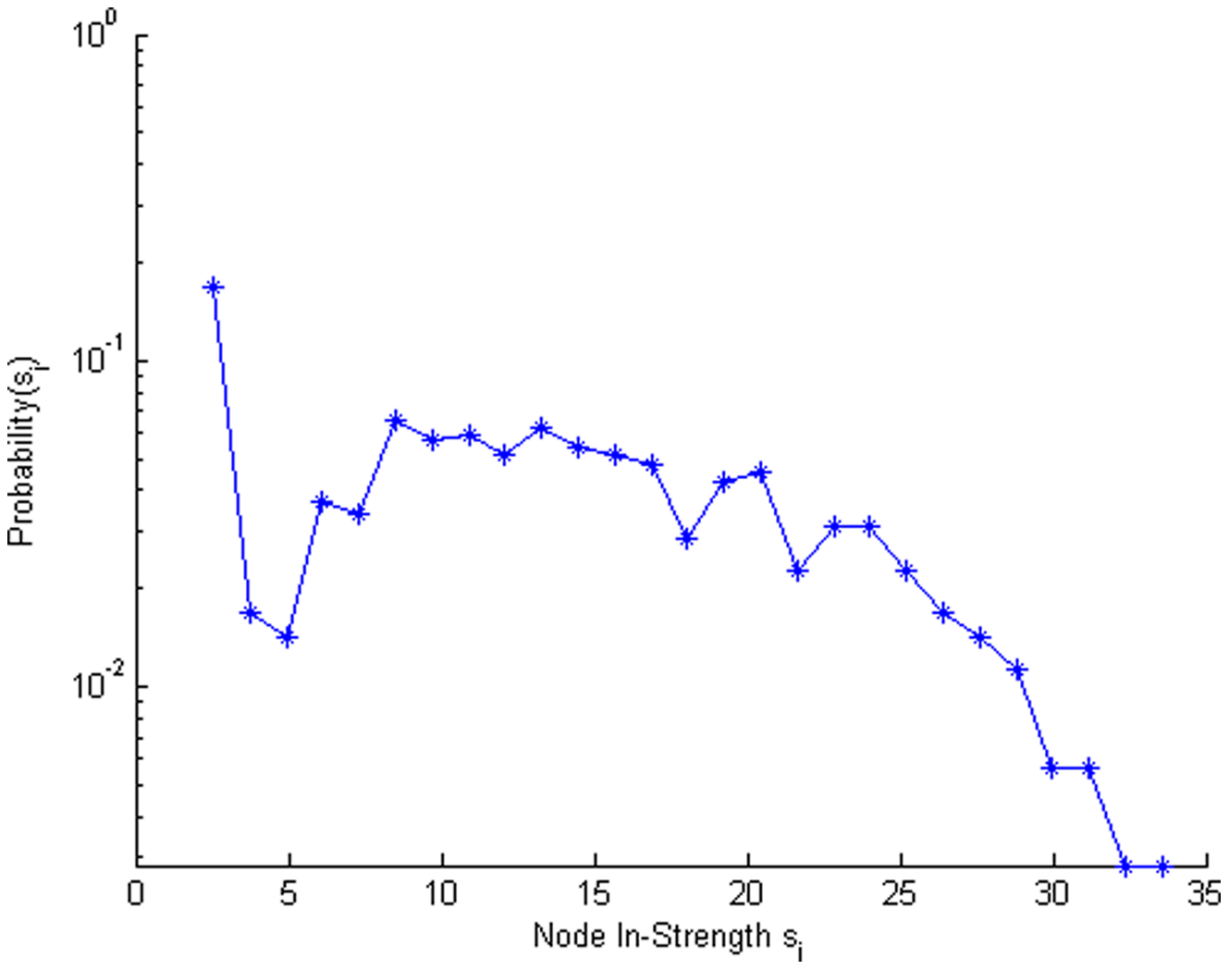
Distribution of the node in-strength values of the individuals in the rural community contact network. Note that the vertical axis has a log scale.

Figure 8 depicts the distribution of the node in-strength metric for the network. It is much less heterogeneous than the node betweenness and link weight distributions as the in-strengths are found rather homogeneous across the nodes. We explored the correlations between the network metrics and various survey responses and found that node betweenness was significantly correlated with age (*r* = −.15, *p*<.01), travel time to work (*r* = .20, *p*<.001), distance to work (*r* = .19, *p*<.001), level of education (*r* = .12, *p*<.05), number of non-family friends contacted weekly (*r* = .51, *p*<.05), and hours away from home each day (*r* = .22, *p*<.001). The outgoing node strength was significantly correlated with age (*r* = −.20, *p*<.001), visiting with more family members outside one’s residence (*r* = .18, *p*<.01), household size (*r* = .12, *p*<.05), travel time to work (*r* = .44, *p*<.001), distance to work (*r* = .45, *p*<.001), compliance risk (*r* = .13, *p*<.05), level of education (*r* = .23, *p*<.001), income (*r* = .17, *p*<.01), having diabetes ( *r* = −.12, *p*<.05), how often one eats out (*r* = .16, *p*<.01), and hours away from home each day (*r* = .50, *p*<.001). Node in-strength was correlated with level of education (r = .12, p<.05) and having had the flu in the past six months (r = −.12, p<.05).

In general, while many of these relationships are not especially strong in terms of effect sizes, it appears that residents with higher levels of education, who have longer commutes, who are younger, with more income, those without diabetes or recent flu-like illnesses, who are away from home more hours each day, and who eat out more often are more likely to be important agents in the network measures that influence the potential spread of epidemics. It is interesting to observe that the younger rural residents are likely the most important agents when considering that rural regions are typically characterized by aging populations. This importance appears to be due to them, the younger persons, spending more time away from home, driving longer to work, visiting more businesses, and in all this, having and visiting more persons outside of their homes. Perhaps, the traditional farmer who rarely visits town and is mostly self-sufficient within his home and immediate neighbors is giving way to a younger generation and changing economy where increased travel and social interaction are increasingly required.

We performed extensive simulations to investigate potential epidemics and the proposed vaccination strategies for the rural contact network representing a sample population from Neosho County. To mimic a realistic epidemic with the stochastic SLIR model, we utilize average values of *λ* ^−1^ = 0.764 days, *µ* ^−1^ = 1.736 days, and *R_0_* = *β/µ* = 1.75 with respective standard deviations of 0.100 days, 0.100 days, and 0.065 [21]–[27]. We explore the hypothetical outbreaks first by simulating 1,000,000 trials of the considered situation (such as without mitigation or with a specific mitigation strategy). For each trial, a triple of (*λ*, *µ*, *R_0_*) is drawn from the three Gaussian distributions with the respective parameters and the outbreak is simulated until it dies out, leaving only susceptible and recovered individuals behind. This first type of experiment attempts to capture the diversity of possible influenza-like outbreaks in the rural community and we use the results of these for numerical comparisons between the different mitigation strategies. However, the irregularity of the parameter values does not yield insightful figures. The second type of experiment we ran was the simulation of sets of 10,000 trials that scan over values *R_0_* to quantify the range of potential outbreaks. In this second type of experiment, we deterministically vary *R_0_*, while *λ* and *µ* are still drawn from their distributions [28], [29].

**Figure 9:**
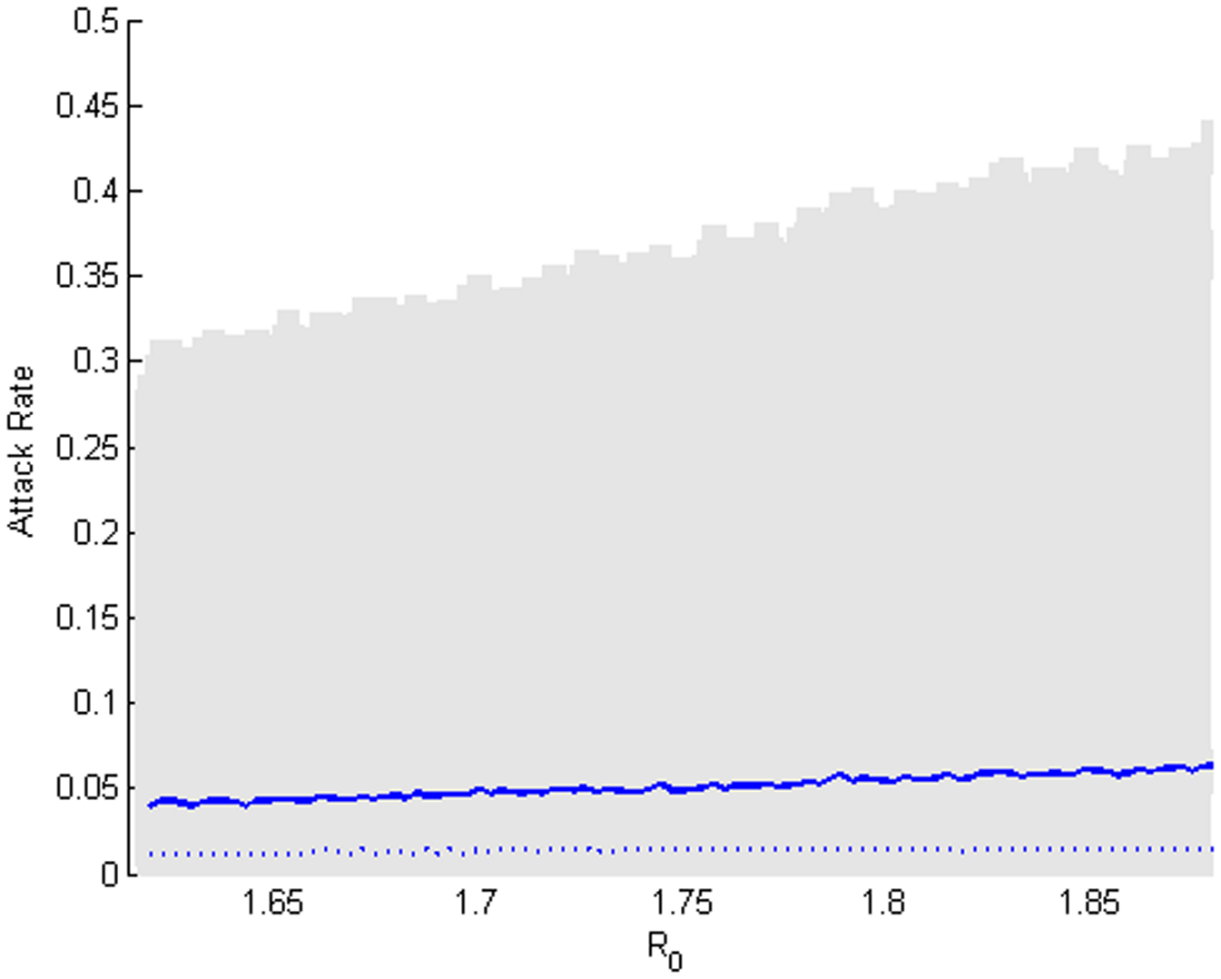
Distributions of attack rates under no mitigation. The distributions of the total cases as a fraction of the considered population over the estimated range of *R_0_* are represented by the medians (dashed blue line), averages (blue line), and 95% confidence interval (grey shaded region). As the infection rate increases, the epidemic size increases in a near-linear manner.

For each simulation, we track the numbers of nodes in each disease state through time as well as the timings of all event occurrences. We capture the total cases, representing this as the attack rate or the fraction of the total population infected, and the duration of each outbreak in days. The duration of an outbreak is the (continuous) time in days from the beginning of the simulation to the recovery (I to R transition) of the last infected node at which point all nodes in the network will either be susceptible or recovered. We define an outbreak as any trial that resulted in at least one secondary infection and present statistics only over the trials successfully demonstrating outbreaks. We simplify the presentation of the results of the second type of experiment by computing and plotting the average and 95% range of the resulting total cases for each group of 10,000 simulations on a single *R_0_* [21]–[23], [25]–[27]. Figure 9 summarizes the distributions of the total cases as a fraction of the population infected (attack rate) in the manner described above. As *R_0_* increases, the epidemic size increases in a near-linear manner. It can be seen that distributions are broad but have low average values. This figure suggests that around 5 percent of the population might on average fall sick during an influenza season, but a few large outbreaks might touch 30–40 percent of the community. It is interesting to observe in figure 9 that the average attack rate varies little over the explored range and the median attack rate varies even less. The regularity of the outbreak distributions for different epidemic strengths is likely due to the strongly connected core of the network and the weakly connected fringes.

**Figure 10:**
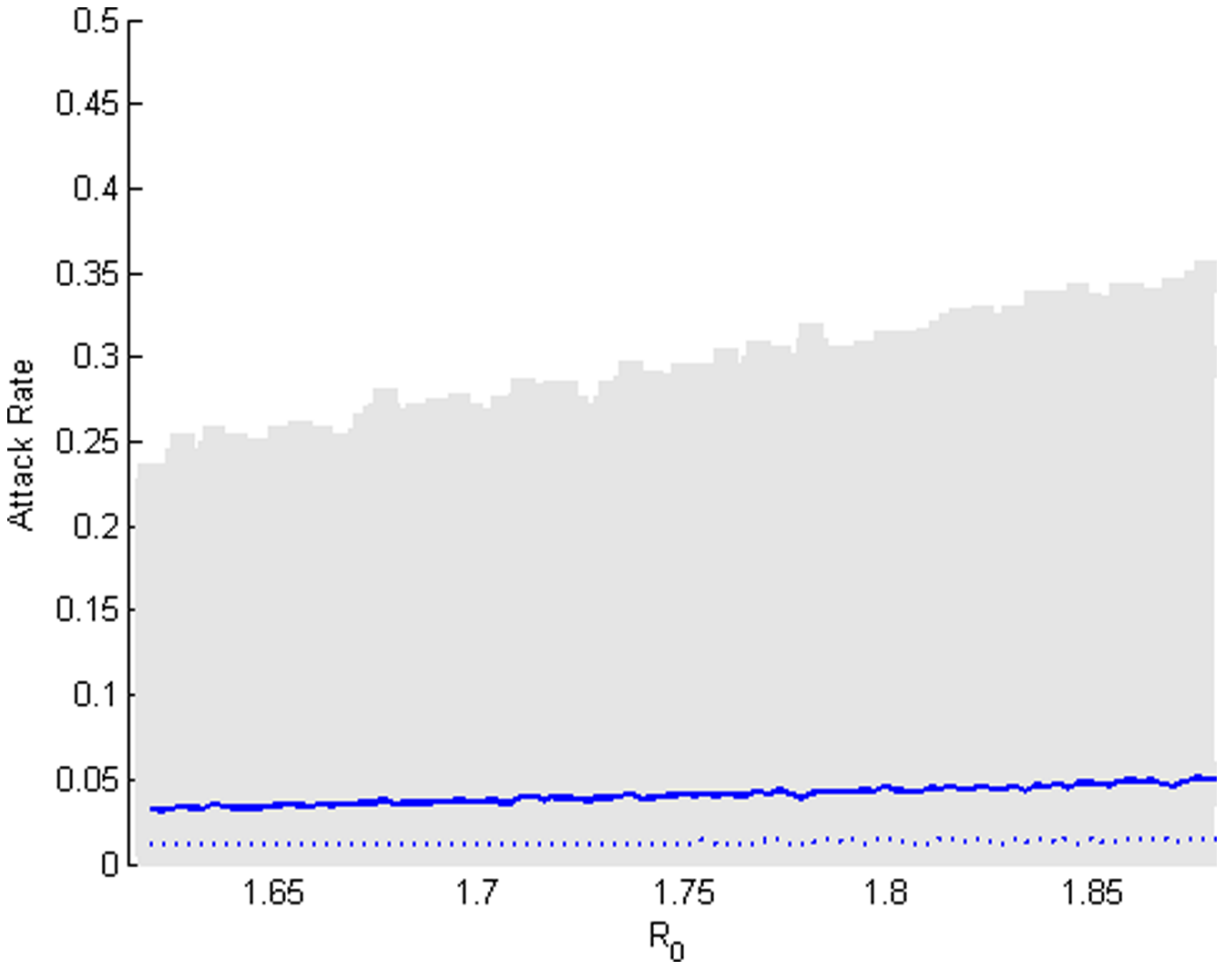
Distributions of attack rates under a random vaccination of 10 percent of the population. The distributions of the total cases as a fraction of the considered population over the estimated range of *R_0_* are represented by the medians (dashed blue line), averages (blue line), and 95% confidence interval (grey shaded region).

We ran seven sets of simulations to consider the seven vaccination strategies described in Section 3 and for each set we ran both types of experiments as described previously. In each trial, we draw a value for vaccine efficacy from a Gaussian distribution with mean of 72.0% and standard deviation of 6.0% to approximate realistic efficacy values [30]–[32]. The first vaccination strategy, the random distribution over the entire population, is the selection of a group of individuals representing 10 percent of the population and administering vaccines prior to the start of an outbreak with the given efficacy. Figure 10 demonstrates the potential reduction in the distributions of outbreaks by random vaccination.

**Figure 11:**
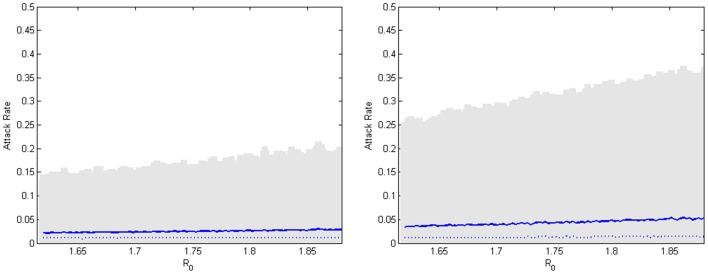
Distributions of attack rates under two mitigation strategies. (Left) Under a node-betweenness-based *individual* targeted vaccination of 10 percent of the population, the distributions of the attack rate over the estimated range of *R_0_* are represented by the medians (dashed blue line), averages (blue line), and 95% confidence interval (grey shaded region). (Right) Under a node-betweenness-based *location* targeted vaccination of 10% of the population, the distributions of the attack rate over the estimated range of *R_0_* are plotted in the same manner.

The three idealistic vaccination strategies select their targets and vaccinate them by rankings determined by the node metrics. The left side of figure 11 captures the reduced epidemic sizes under an individual targeting strategy which uses node betweenness to select the individuals. For a realistic targeting of a distribution location, the right side of figure 11 captures the potential reductions in the epidemic sizes under the node-betweenness-based location targeting strategy. The location-based strategies are intuitively less successful than the individual targeting methods, but they represent much more feasible options for an administrative intervention.

**Figure 12:**
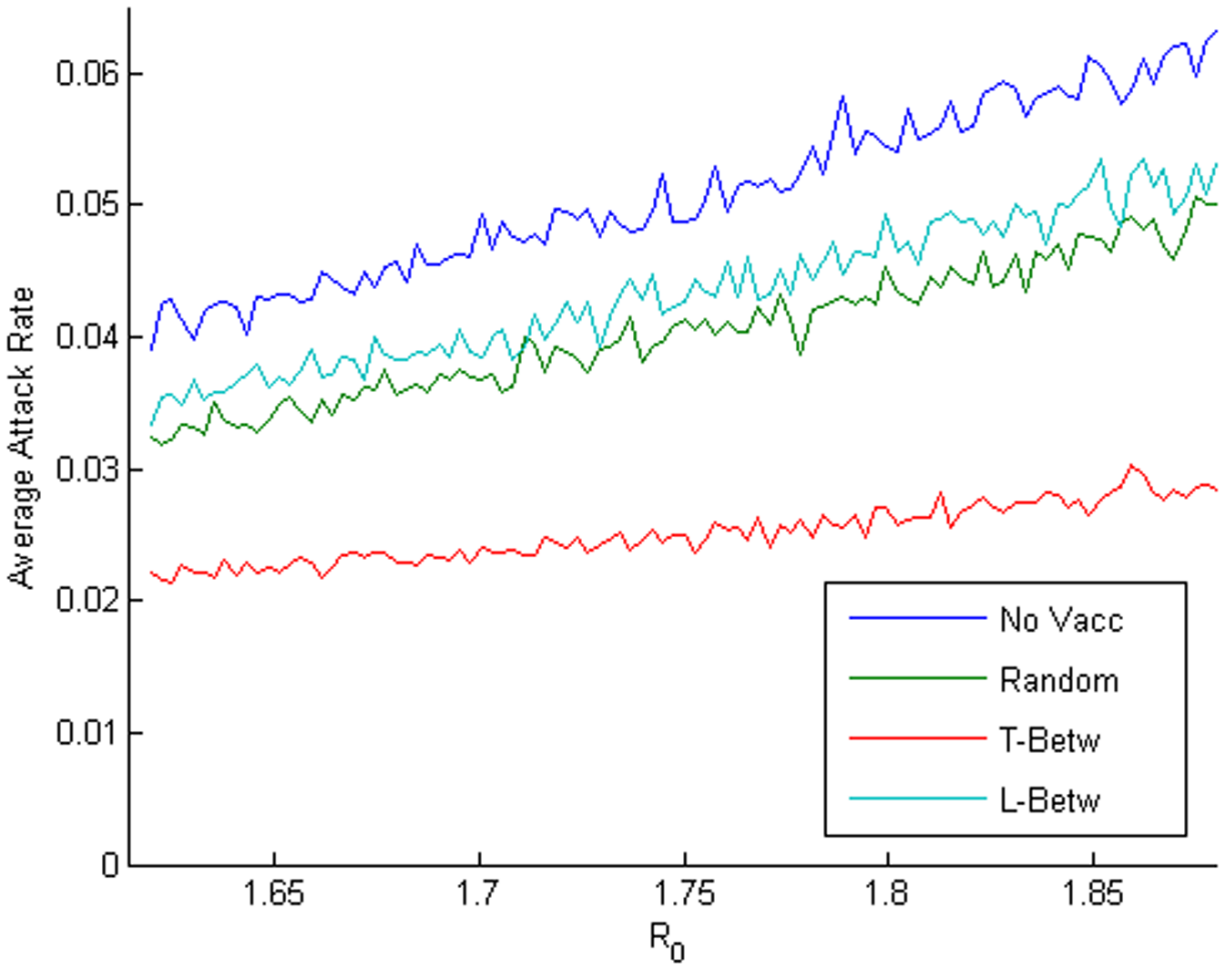
Comparison of average attack rates for different mitigation strategies. The comparison of no mitigation (No Vacc, blue line), a random vaccination of 10 percent of the population (Random, green line), node-betweenness-based *individual* targeted vaccination (T-Betw, red line), and a node-betweenness-based *location* targeted vaccination (L-Betw, teal line) is represented by their respective average attack rates over the estimated range of *R_0_*.

A brief comparison of the results shown in figures 9, 10, 11 can be seen in figure 12. Figure 12 plots the average attack rates of the three strategies and the case of no vaccination. It can be seen that the situation of no vaccination results in the highest average attack rates, while the individually targeting strategy results in the lowest average attack rates and the remaining two strategies appear similar in an intermediate level of effectiveness. The distributions of the epidemic durations are not shown as they did not vary as *R_0_* varies. We summarize the comparison of the different vaccination strategies under the first type of experiments in table 1, which describes the distributions of attack rates and epidemic durations in days (in italics) by their averages, medians and 95% confidence intervals. It is immediately interesting to notice that each of the vaccination strategies reduces the average epidemic duration, some by as much as 20 percent on the average value. In table 1, it can be seen that the individual targeting methods have the highest average results, but among the feasible methods, the location-based targeting using the node betweenness metric is the most successful at reducing the total cases on average. The node betweenness also provides the best metric for the individual targeting strategies.

**Table 1:** The attack rates of different strategies and the duration of the epidemic outbreaks in days (*in italics*) shown by the averages, medians, and confidence intervals of the distributions.

	Average	Median	95% CI
No Vaccination	0.0512	0.0142	(0.0057, 0.3088)
	*10.7200*	*7.3066*	*(1.6101, 35.6527)*
Random Vaccination	0.0407	0.0113	(0.0057, 0.2493)
	*9.9031*	*7.0369*	*(1.6190, 32.4859)*
Targeted Betweenness	0.0251	0.0113	(0.0057, 0.1388)
	*8.3638*	*6.5215*	*(1.5960, 25.2766)*
Targeted In-strength	0.0324	0.0113	(0.0057, 0.1955)
	*9.1509*	*6.7562*	*(1.6046, 29.4892)*
Targeted Out-strength	0.0261	0.0113	(0.0057, 0.1445)
	*8.4930*	*6.5795*	*(1.6046, 25.8235)*
Location Targeted Betweenness	0.0433	0.0113	(0.0057, 0.2635)
	*10.1597*	*7.1323*	*(1.6073, 33.5715)*
Location Targeted In-strength	0.0433	0.0113	(0.0057, 0.2635)
	*10.1652*	*7.1454*	*(1.6231, 33.4379)*
Location Targeted Out-strength	0.0434	0.0113	(0.0057, 0.2635)
	*10.1558*	*7.1178*	*(1.6153, 33.5992)*

The last column of table 2 displays the cases prevented per vaccine distributed. The value cases prevented per vaccine has an intuitive benchmark of the average vaccine efficacy at 0.72. If a vaccination strategy is very efficient at stopping an outbreak, then we can expect the average number of cases prevented per vaccine to be higher than the typical efficacy of the vaccine. On the other hand, if a vaccinated trial is resulting in an average number of cases prevented per vaccine that is less than the typical vaccine efficacy, it doesn’t necessitate that the vaccination strategy will perform poorly in all situations. In general this situation implies that the vaccines are being given to individuals who usually aren’t being infected and therefore they made little use of the vaccine in that set of trials. This could arise from either a poor vaccine distribution strategy or from a distribution of vaccines that is larger in size than a typical outbreak. When we have a strong outbreak, the vaccines are almost surely going to be a necessary measure, whereas in a weaker outbreak, most of the population will not be infected and extra vaccines will be “unused” with respect to preventing new cases. Notice that for the first type of experiment when we are not considering any vaccination the epidemic impacts roughly 5.1 percent of the population while the number of vaccines distributed is sufficient for 10 percent of the population.

**Table 2:** The probabilities of an outbreak occurring under the eight different scenarios considered and the cases prevented per vaccine distributed.

	Probability of outbreak	Cases Prevented Per Vaccine
No Vaccination	0.3928	—
Random Vaccination	0.3783	0.1065
Targeted Betweenness	0.3742	0.2638
Targeted In-strength	0.3623	0.1898
Targeted Out-strength	0.3768	0.2537
Location Targeted Betweenness	0.3778	0.0797
Location Targeted In-strength	0.3783	0.0795
Location Targeted Out-strength	0.3776	0.0790

An outbreak is defined as the occurrence of at least one secondary infection from the initial infected node.

Table 2 lists the probabilities of outbreaks occurring under each of the eight scenarios considered. Of particular interest is that the targeted in-strength approach, having the lowest probability of an outbreak, has a higher average attack rate and longer average epidemic duration than the other targeted strategies.

## Discussion

From the network analysis, we observed that the rural contact structure displays a significant amount of heterogeneity in the considered metrics. This heterogeneity suggests that the small number of nodes having the highest values of each metric might present strategic sub-populations for mitigation objectives. The rural contact network also contained a relatively disease-resistant sub-population due to their poor level of connectivity and location on the “fringes” of the rural community network. From statistical correlations, it appears that residents with higher levels of education, who have longer commutes, who are younger, with more income, those without diabetes or recent flu-like illnesses, who are away from home more hours each day, and who eat out more often are more likely to be important players in the according to the network metrics that influence the potential spread of infectious diseases.

In the data collected from the rural survey, there remain significant limitations. Although the survey presents a variety of types of locations such as schools, restaurants, libraries, and public attractions, the data does not sufficiently capture the information regarding household interactions. It was not feasible to anonymously identify individual households and which survey respondents visited them with the resources at our disposal. The lack of information regarding young respondents and household interactions remains a strong limitation in the characterization of this community and the following epidemic study on the rural contact network.

For vaccine distribution we considered seven strategies, but only four are reasonably feasible for local administrators to implement, those being the random distribution across the population and the three location-based distributions. The traditional targeted groups for distribution such as the health-care personnel, the very young (6–59 months), the elderly (50 years or older), pregnant women, those with chronic health issues, and American Indians are not completely identifiable from our survey results [33]. We could identify respondents by age range, but occupation and maternity status are transitory positions and were not explored by the survey. The global random distribution of vaccines gives a simplest method to compare the other vaccination methods to. The location-based methods are indicative for anonymously targeting subpopulations, not only for vaccination campaigns, but also for educational outreach to encourage social responses such as adoption of preventative health practices.

Interestingly, using the network metrics to select locations does not necessarily produce intuitive results. The restaurant chosen to represent locations that are frequented by nodes with high node outgoing node strength (as it had the highest average value) had less than one-third of the survey respondents frequenting it than some of the more popular restaurants in the region. Although diseases are partially mitigated, there is a limit to the reduction that can be observed in the total cases for the strongest diseases due to the resource limitation. Therefore when considering limited-resource vaccine distribution, local administrators should probably follow the traditional priority schedule. However, the identification of the critical locations would be useful for preventative education efforts, real-time epidemic alerts, and emergency resource distribution.

The results of this analysis are intended to help guide responses to a rural epidemic threat. With this, responders can explore the theoretical impacts that might be had from a limited-resource vaccine distribution by exploring various locations for distribution. Social behavior and human interaction (contact) are not exact sciences, so the theoretical mitigation results should be considered possibilities and aspirations rather than deterministic outcomes for any rural county or town.

## Conclusions

Starting with a survey of a rural community, demographics were analyzed and an estimation of the social contact structure was built. This network was measured and the metrics were correlated with various demographics from the survey. Through the use of an exact model of a stochastic SLIR Poisson process, we have characterized a typical influenza-like outbreak in the community and investigated vaccination strategies. When considering resource-limited vaccine distribution strategies, we identified critical locations for ethical targeting subpopulations with the goal of effective disease prevention. Our aspiration is that this analysis will be a valuable resource for both the rural community on which this study focused, and also for several similar communities in the region.
